# Favorable outcome of empagliflozin treatment in two pediatric glycogen storage disease type 1b patients

**DOI:** 10.3389/fped.2022.1071464

**Published:** 2022-11-23

**Authors:** Zufit Hexner-Erlichman, Maria Veiga-da-Cunha, Yoav Zehavi, Zahava Vadasz, Adi D. Sabag, Sameh Tatour, Ronen Spiegel

**Affiliations:** ^1^ Department of Pediatrics B, Emek Medical Center, Afula, Israel; ^2^ Genetic Institute and Center for Rare Diseases, Emek Medical Center, Afula, Israel; ^3^ Metabolic Research Group, de Duve Institute and UCLouvain, Brussels, Belgium; ^4^ Ruth and Bruce Rappaport Faculty of Medicine, Technion, Haifa, Israel; ^5^ The Proteomic and Clinical Flow Cytometry Unit, Bnai-Zion Medical Center, Haifa, Israel; ^6^ Pediatric Gastroenterology Unit, Emek Medical Center, Afula, Israel

**Keywords:** glycogen storage disease type 1B, neutropenia, neutrophil dysfunction, inflammatory bowel disease, empagliflozin

## Abstract

**Background:**

Glycogen storage disease type 1b (GSD1b) is an ultra-rare autosomal recessive disorder, caused by mutations in *SLC37A4* gene. Affected patients present with episodes of fasting hypoglycemia and lactic acidosis, hepatomegaly, growth retardation, hyperlipidemia and renal impairment. In addition, patients present neutropenia, neutrophil dysfunction and oral, and skin infections as well as a significant predisposition to develop inflammatory bowel disease (IBD). Low neutrophil counts and function is related to the toxic accumulation of 1,5-anhydroglucitol-6-phosphate (1,5-AG6P). Recently, several reports have shown that off-label treatment with empagliflozin (EMPA), an inhibitor of the renal glucose transporter SGLT2, decreased blood 1,5-anhydroglucitol (1,5-AG), and neutrophil 1,5-AG6P, thus resulting in a new therapeutic option for neutropenia and neutrophil dysfunction in patients.

**Methods:**

Off-label treatment with EMPA was established in two GSD1b patients after signed informed consent. The patients were followed clinically. We monitored neutrophil counts and function, 1,5-AG levels in plasma and its renal clearance before and during EMPA treatment.

**Results:**

A 17 year-old girl who had long standing oral ulcers and developed IBD, requiring systemic steroid and regular granulocyte colony-stimulating factor (GCSF) therapy and an 8 year-old boy who had steady non healing oral lesions were treated with empagliflozin during 18–24 months. Treatment led to increase of neutrophil counts and function with substantial clinical improvement. This included remission of IBD in the first patient which allowed to discontinue both GCSF and steroid therapy and resolution of oral lesions in both patients. The concentration of 1,5-AG in blood was greatly decreased within two weeks of treatment and remained stable thereafter.

**Conclusions:**

Repurposing of empagliflozin to treat neutropenia in two GSD1b patients was safe and resulted in the urinary excretion of 1,5-AG, the normalization of neutrophil function, and a remarkable improvement of neutropenia-related clinical traits. We showed for the first time that empagliflozin increases concomitantly the renal clearance of both 1,5-anhydroglucitol and glucose in GSD1b patients.

## Introduction

Glycogen storage disease type 1 (GSD1) [MIM#232220] is a metabolic disorder that results from a defect in the final step of glycogen breakdown. It has two subtypes; GSD1a, caused by a deficiency in glucose-6-phosphatase (G6Pase), an integral membrane phosphatase, with its catalytic site facing the lumen of the endoplasmic reticulum (ER) and GSD1b, caused by a deficiency in G6PT, the dedicated glucose-6-phosphate transporter of the endoplasmic reticulum (1). GSD1b is an ultra-rare autosomal recessive disorder caused by bi-allelic mutations in the *SLC37A4* gene and is less common compared with GSD1a (1).

GSD1a usually presents in the infantile period with severe fasting hypoglycemia and lactic acidosis. In addition, patients develop hepatomegaly, growth retardation, delayed puberty, doll-like facies, hypotrophic muscles, enlarged kidneys, hyperuricemia and hyperlipidemia with mainly hypertriglyceridemia. Recurrent episodes of life-threatening hypoglycemia and lactic acidosis may occur during fasting and trivial febrile illnesses (2, 3). In addition, GSD1b is characterized by variable neutropenia and neutrophil dysfunction that may require treatment with granulocyte colony-stimulating factor (GCSF) to improve neutrophil counts. This can result in recurrent infections, including oral mucositis, severe dental carries, ano-urogenital infections and significant predisposition to develop inflammatory bowel disease (IBD), often despite the GCSF treatment (4–7).

In 2019 Veiga-Da-Cunha and colleagues discovered the essential role of G6PT in neutrophils, which explains neutropenia in GSD1b and Glucose-6-Phosphatase Catalytic Subunit 3 (G6PC3)-deficient patients (8). Accordingly, in neutrophils, the function of G6PT is to transport 1,5-anhydroglucitol-6-phosphate (1,5-AG6P), a phosphoric ester very similar to glucose-6-phosphate, into the ER. In neutrophils deficient in G6PT or G6PC3, the accumulation of 1,5-AG6P results in the inhibition of hexokinase, the main glucose phosphorylating enzyme that catalyzes the first step of glycolysis. This is especially crucial in neutrophils in which glucose-6-phosphate serves as the main source for energy production in glycolysis and nicotinamide adenine dinucleotide phosphate (NADPH) in the pentose phosphate pathway, essential for an active respiratory burst. Consequently, a defect in the production of glucose-6-phosphate leads to a deficit in energy production, to neutrophil dysfunction and subsequent apoptosis (8, 9).

Empagliflozin (EMPA), an inhibitor of SGLT2, the renal sodium-glucose co-transporter 2, widely used in the treatment of type 2 diabetes by decreasing glucose reabsorption in the kidney was recently shown to also inhibit 1,5-anhydroglucitol (1,5-AG) renal reabsorption, thus reducing its concentration in blood and the consequent cellular accumulation of toxic 1,5-AG6P in neutrophils (8, 10). Subsequently, several case reports have shown beneficial effects of EMPA treatment on neutropenia in GSD1b and G6PC3-deficient patients. This improved neutrophil counts and function resulting in clinical improvement and in some cases discontinuation of GCSF treatment (10–14).

In this report we describe the clinical course of two pediatric GSD1b patients who were treated with EMPA for 18 and 24 months respectively. In addition, we report long term blood 1,5-AG monitoring, neutrophil counts and function and glucose and 1,5-AG renal clearance before and after EMPA treatment in our patients.

## Materials and methods

### Assessment of plasma levels and renal clearance of 1,5AG

1,5-AG was quantified by liquid chromatography—mass spectrometry (LCMS) in plasma and urine, urinary glucose in neutralized perchloric acid extracts was measured spectrophotometrically and urinary clearance for 1,5-AG and glucose were all determined as previously described (9, 15). Practically, 1,5-AG in plasma and in 24 h urine collections were extracted by adding respectively 4 µl of plasma or 2 µl of urine to 91 or 93 µl of a solution containing 81% methanol, 10% H2O and 9% chloroform and 5 µl of deuterated 30 µM 2-[D]-1,5AG (m/z = 164.0674), which was used as an internal standard to allow estimation of plasma or urinary 1,5-AG (m/z = 163.0612). Absolute concentrations were determined by comparing the integrated extracted ion chromatograms corresponding to 1,5-AG with those of the internal standard (10).

Urinary glucose was measured in a 5% perchloric acid extract from an aliquot of a 24 h urine collection, which was neutralized by careful addition of the required volume of a 3 M KOH/KHCO3 solution. After removing the insoluble potassium perchlorate by centrifugation (5 min at 12,000g at 4°C) the glucose in this extract was measured spectrophotometrically using an hexokinase/glucose-6-phosphate dehydrogenase coupled assay run at 37°C. The assay mixture (1 ml in an assay cuvette) contained 100 mM Hepes buffer at pH 7.2, 10 mM MgCl2, 40 mM KCl, 1 mg/ml BSA, 2 mM ATP-Mg2+, 0.6 mM NAD+, 6 µl of neutralized urine extract and a nonlimiting amount of glucose-6-phosphate dehydrogenase. After 10 min, we added a nonlimiting amount of yeast hexokinase. The consequent increase in absorbance at 340 nm is directly proportional to the concentration of glucose (16).

### Neutrophil function analysis

Neutrophil respiratory burst was measured directly in fresh whole blood using PHAGOBURST™ assay (Celonic Deutschland GmbH, Heidelberg, Germany) by flow cytometry, focusing specifically on granulocytes. Venous blood was collected using sodium–heparin tubes; samples were stored at room temperature and the analyses were performed within 24 h of sampling, according to the manufacturer's instructions: samples of whole blood were stimulated with either phorbol 12-myristate 13-acetate (PMA) as high stimulus, or N-formyl-MetLeuPhe (fMLP) as low stimulus, or pre-opsonised *E. coli* bacteria (1–2 × 10^9^/ml) as particulate bacterial stimulus. A sample without stimulus served as negative background control.

Samples were incubated in a water bath at 37°C for 10 min before adding 20 µl of substrate solution and continuing the incubation for an additional 10 min. The reaction was stopped by adding the lysing solution, which removed erythrocytes and partially fixed leukocytes. After a washing step, DNA staining was performed to exclude cell debris and aggregation artifacts from bacteria. The samples were then assayed using Navious EX Flow Cytometer (Beckman Coulter, USA). Data were analyzed using Kaluza software (Beckman Coulter, USA) and the values were reported as the percentage of cells exhibiting fluorescent oxidized dihydrorhodamine 123 (DHR) of the total events and the average intensity of oxidative burst was estimated by the mean fluorescence intensity (MFI) per cell.

## Results

### Case reports

Patient 1 (PT1): This patient is 17.5 year-old female, the fourth offspring of healthy first-degree cousins of Arab Muslim origin. Her sister died at the age of 9 years due to systemic complications of GSD1b and poor compliance to conventional treatment. Genetic analysis identified the previously described homozygous pathogenic variant c.466G > A (p.Gly149Glu) in *SLC37A4* gene as the cause of the sister's disease (17). Our patient presented initially at the age of 2 months with severe hypoglycemia, lactic acidosis and hepatomegaly and based on family history she was found homozygous to the c.466G > A pathogenic variant in *SLC37A4* gene confirming her clinical diagnosis of GSD1b.

**Figure 1:**
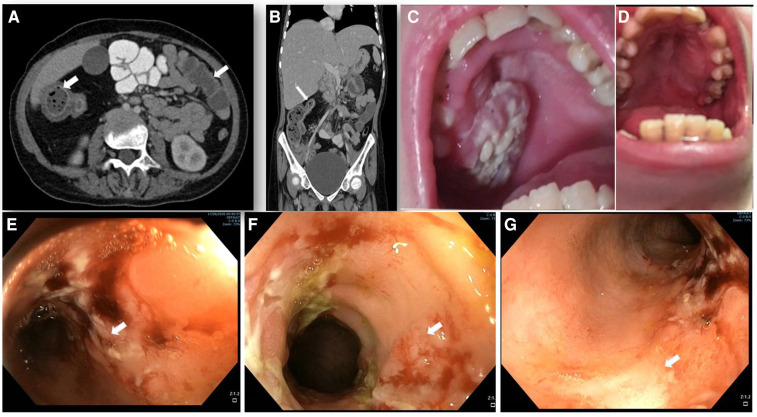
Abdominal imaging, endoscopy and clinical features. (**A,B**) abdominal computed tomography (CT) with contrast enhancement. (**A**) horizontal plane displaying the increased wall thickness of the ascending and transverse colon (arrows). (**B**) coronal plane demonstrating stricture of the transverse colon lumen (arrow). (**C**) oral sores and lesions before EMPA treatment and (**D**) its resolution after 12 months of treatment. (**E–G**) colonoscopy showing IBD-like colitis involving descending (**E**), ascending (**F**) and transverse (**G**) colon with severe inflammation, fibrin plaques, and mucosal ulcers (white arrows) and skip lesions with areas of Normal mucosa.

The family was only partially compliant with the dietary recommendations, in particular with overnight feeding either by nasogastric tube or gastrostomy. As a result, the patient developed various GSD1b-related complications including long standing hyperlipidemia with extremely elevated serum triglyceride levels ranging between 1,000 and 2,500 mg/dl (desirable <150 mg/dl, borderline-high 150–199 mg/dl, high 200–499 mg/dl extreme ≥500 mg/dl), hypercholesterolemia, severe growth retardation, delayed puberty, massive hepatomegaly, osteopenia, nephromegaly with proteinuria due to glomerulopathy (for which treatment with inhibitor of angiotensin converting enzyme 5 mg/day was initiated) and renal tubular dysfunction. In addition, she had several episodes of metabolic crises requiring intensive care admissions due to life-threatening hypoglycemia and lactic acidosis. Given her GSD1b, she also had fluctuating neutropenia and neutrophil dysfunction that resulted in recurrent oral and skin infections, extensive dental carries, and long standing oral aphthous lesions (Figure 1C). She started treatment with subcutaneous GCSF at the age of 14.5 years (5 mcg/kg/twice a week) due to persistent neutropenia and persistent non-healing oral ulcers.

**Figure 2:**
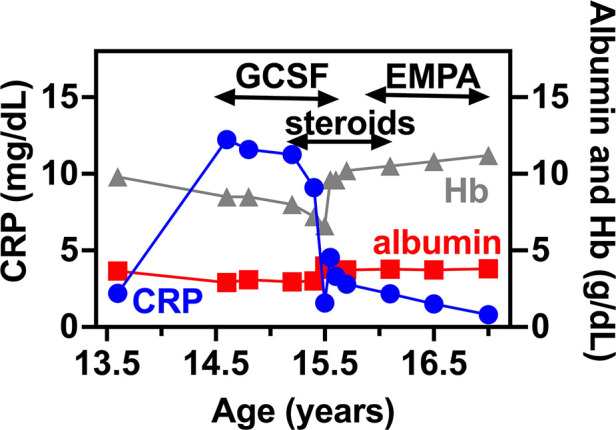
IBD related biomarkers for PT1. CRP, serum albumin and hemoglobin levels are shown in relation to PT1 IBD disease course. In specific, GCSF, systemic steroids and EMPA treatment are shown as upper bars.

At 15.3 years she presented with progressive fatigue, abdominal distention, weight loss, bloody diarrhea and severe anemia. Her fecal calprotectin level was 1,470 mcg/g (normal range 0–100 mcg/g) and she was diagnosed with Crohn's-like colitis with sparing of the small intestine and rectum (Figures 1A,B,E–G). She started treatment with systemic steroids (oral prednisone 25 mg/day with slow tapering down), sulfasalazine (1 gram/day) and specific nutritional dietary management (Modulen®) in addition to increasing GCSF injections to every other day. Clinical improvement was achieved with gradual gain of weight, normal stools, and significant decrease of CRP levels, albumin, and hemoglobin concentrations as well as remarkable reduction of her fecal calprotectin (a reliable marker of disease activity) to 110 mcg/g (normal range 0–100 mcg/g). At the age of 15.9 years, off-label oral treatment with EMPA was started in order to improve neutrophil count and function and associated clinical features including better control of her IBD. Initially, she was admitted for inpatient monitoring of glucose levels, vital signs, urinary output, renal function, hemoglobin concentration levels, absolute neutrophil count (ANC) and evaluation of potential adverse side-effects. Within two days we increased the dose to 0.5 mg/kg/day with no adverse side-effects which allowed safe discharge and out-patient follow-up of glucose levels. Since then, the patient experienced complete normalization of her ANC allowing discontinuation of GCSF therapy one month after initiating EMPA treatment. In addition, her IBD improved with gradual discontinuation of systemic steroids and stabilization of serum albumin and CRP levels and hemoglobin levels (Figure 2) as well as fecal calprotectin. She continued treatment with oral sulfasalazine 1 g/day.

Since the age of 16.9 years the patient had recurrent episodes of acute pancreatitis that were presumably caused by long-standing severely elevated triglyceride levels. Despite conservative supportive treatment and dietary modifications as well as oral fibrate therapy and omega-3 fatty acids supplementation, serum triglycerides remained elevated and the patient is gradually developing chronic pancreatitis but with pancreatic exocrine and endocrine sufficiency.

**Figure 3:**
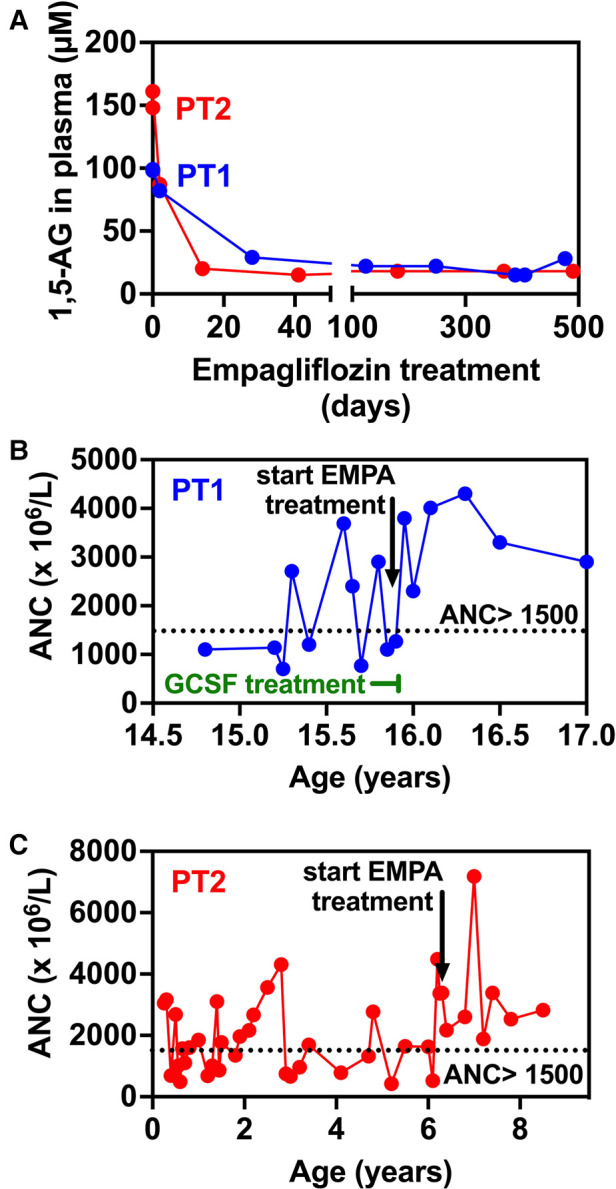
Evolution of 1,5-anhydroglucitol and neutrophils counts in blood before and during empagliflozin (EMPA) treatment for PT1 and PT2. (**A**) 1,5-AG present in plasma was measured by LC-MS analysis in blood samples from PT1 and PT2 taken before and during treatment. (**B,C**). The time-line shows that in the case of both patients, following the initiation of EMPA treatment, ANC remain above 1,500 × 10^6^/l (despite the end of GCSF treatment in PT1—panel B) indicating the absence of neutropenia episodes (ANC < 1,500 × 10^6^/l).

At the age of 17.5 years, the patient has multiple disease complications including severe growth retardation, delayed puberty, severe hypertriglyceridemia, hypercholesterolemia, renal glomerular and tubular dysfunction, osteopenia and evolving chronic pancreatitis. She manages to keep normal glucose level with no hypoglycemic events (continuous capillary glucose monitoring) but has chronic mild serum lactate elevation suggesting suboptimal metabolic control although she did not suffer significant metabolic crises for several years. She has been taking EMPA for 18 months with regular monitoring of serum 1,5-AG levels which decreased substantially within few days after initiating therapy (Figure 3A) and have remained low. Her ANC levels continued within normal levels (Figure 3B) and her IBD is controlled with no flare ups (remissions) and with only sulfasalazine maintenance but no systemic steroids. In addition, her oral ulcers and chronic stomatitis recovered completely (Figure 1D). After 18 months, we increased empagliflozin levels to 0.65 mg/kg/day due to mild elevation of serum 1,5-AG, increase in stool frequency although no diarrhea and mild decrease of ANC while still within normal range.

**Figure 4:**
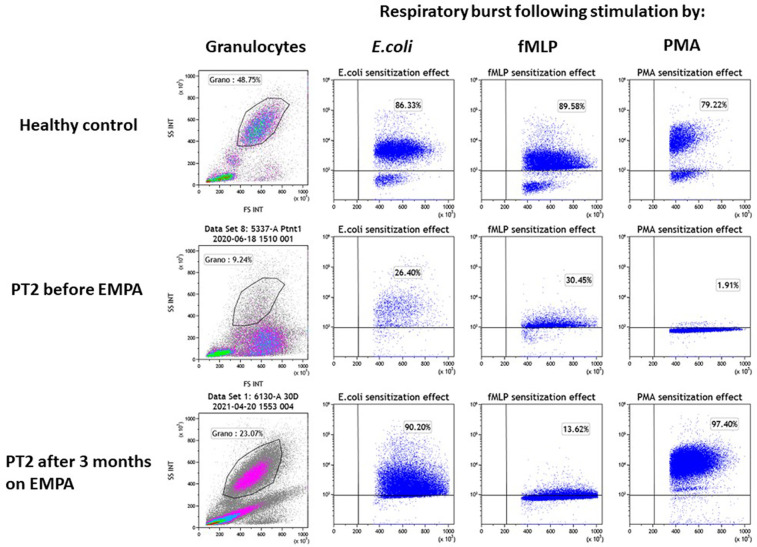
Evaluation of the impact of empagliflozin treatment on neutrophil function. Neutrophil function was evaluated for PT2 by measuring the respiratory burst capacity of granulocytes before and after EMPA treatment. Granulocyte abundance is shown on the left column as the percentage of leukocytes measured by flow cytometry. The impact of *E.coli*, fMLP and PMA (second, third and fourth columns from left) is shown on the stimulation of the respiratory burst in blood samples from a healthy control (top row) and PT2 before (middle row) and after 3 month on EMPA treatment (bottom row). The assay shows normalization of neutrophil's function and number after 3 months of EMPA therapy.

Patient 2 (PT2): This 8.5 year-old boy is the fifth offspring of healthy first-degree cousins of Arab Muslim origin but with no familial relation to PT1. A first-degree cousin of PT2's mother and father, homozygous for the pathogenic variant c.466G > A, in *SLC37A4* gene, which is the same as the one found in PT1, died at the age of 22 years due to GSD1b. Interestingly, he also had poor compliance and died as a result of several late complications of his GSD1b disease including chronic pancreatitis and consequent pancreatic failure necessitating endocrine and exocrine replacement therapy (17). PT2 presented at the age of 3 months during febrile illness with hypoglycemia, lactic acidosis and hepatomegaly and elevated transaminases levels. Given the family history, genetic testing confirmed the clinical diagnosis of GSD1b. Consequently, he started a diet based on frequent meals of hydrolyzed formula, with nocturnal feedings administered *via* gastrostomy tube that was placed at the age of 6 months which allowed for effective intervention during metabolic decompensations or febrile illnesses including hospital admissions when required. At the age of 1 year uncooked cornstarch was added to the diet in order to better maintain normoglycemia. Glucose levels were monitored using continuous capillary glucose sensor with good metabolic control. This management resulted in normal growth, normal daily glucose levels and the prevention of the onset of complications that are common in GSD1b patients. In particular, renal functions were regularly assessed and were normal with no evidence of proteinuria. In line with good metabolic control cholesterol levels are normal and serum triglyceride levels (200–400 mg/dl) are only mildly elevated. PT2 had only one intensive care admission due to hypoglycemia and metabolic acidosis following a gastroenteritis episode. He had transient neutropenia and neutrophil dysfunction, manifested by recurrent episodes of gingivitis, oral ulcers and dental carries, together with skin infections that included poor and very slow healing following the insertion of the gastric tube for feeding. Subcutaneous GCSF was administered for limited days (a total of 3 courses with no longer than 5 days for each course) during significant infections. At the age of 6.3 years, off-label EMPA treatment was started in hospital with close monitoring of glucose levels, vital signs, renal functions and urinary output. Once tolerated we increased the dose on the third day to 0.5 mg/kg/day and continued treatment and out-patient follow-up with continuous capillary glucose monitoring. Clinical improvement was noted, as oral sores and aphthous lesions resolved together with complete restoration of neutrophil count and function (Figures 3C, 4). Continuous glucose monitoring confirmed normoglycemia and excluded hypoglycemic episodes. All together, EMPA was safe and was not associated with adverse side-effects. At the age of 8.5 years, the patient has been treated with EMPA for already 2 years (0.5 mg/kg/day) with a substantial and stable decrease in the level of 1,5-AG in blood (Figure 3A). His oral lesions resolved completely allowing good dental and oral hygiene.

### ANC levels and neutrophil function

Prior to EMPA treatment, both patients had fluctuating ANC, ranging between normal levels and moderate neutropenia. Of note, these levels were somewhat biased since blood counts were regularly obtained during medical referrals when patients were stressed (febrile illness, metabolic decompensation etc.) and we anticipate ANC during unstressed conditions to be even lower. As expected, ANC increased following EMPA administration in both patients and have remained within normal levels since (18 months for PT1, 24 months for PT2—Figures 3B,C). Accordingly, GCSF treatment in PT1 was discontinued after 30 days following the initiation of EMPA treatment.

### Neutrophil function

In both patients, the stimulation of the respiratory burst with *E. coli* was severely impaired before EMPA treatment (PT1–42.2%; PT2–26.4%; healthy controls >75%) and was normalized when tested 3 months after treatment with EMPA (PT1–90.2%; PT2–86.6% respectively). Stimulation of the respiratory burst with PMA was equally deficient in granulocytes from both patients (PT1–7.3%; PT2–1.9%; healthy controls >80%) and was normalized after EMPA treatment (PT1–95%; PT2–97%) (Figure 4). Stimulation with fMLP (low stimulant) was normal before and after EMPA in both patients (normal range >10% in this kit according to manufacturer).

### Plasma 1,5-AG levels

Following EMPA therapy, 1,5-AG in plasma decreased significantly within two weeks and remained stable thereafter in both patients. Before treatment, initial 1,5-AG baseline levels were lower in PT1 (100 µM) compared to PT2 (160 µM), while on EMPA, they were stably reduced to approximately 20 µM, in agreement with the clinical improvement seen for both patients. After 18 months treatment, following a slight increase in 1,5-AG levels in PT1, EMPA dosage was increased to 0.65 mg/kg/day (Figure 3A).

### Glucose and 1,5-AG renal clearance

**Figure 5:**
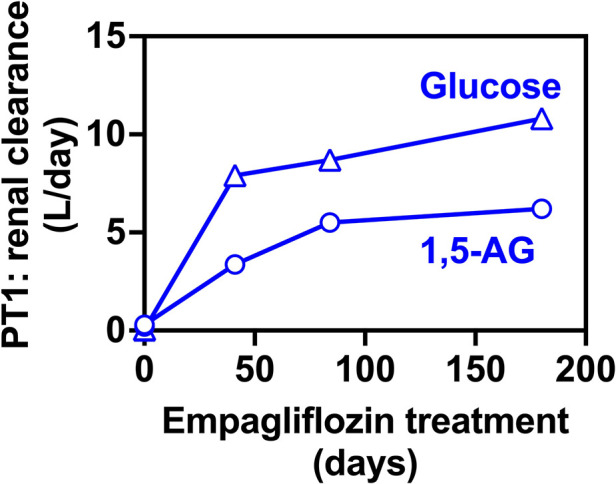
Renal clearance for 1,5-anhydroglucitol and glucose before and during EMPA treatment for PT2. Renal clearance of 1,5-AG increases in parallel to the glucose clearance in 24-hr urine collections for PT2, indicating that glucosuria prevents the renal uptake of 1,5-AG and results in its urinary excretion.

We have also evaluated glucose and 1,5-AG renal clearance. This was done by collecting 24 h urine samples only in PT1 who was more cooperative. We collected urine samples before and after EMPA treatment which nicely demonstrated that renal clearance of 1,5-AG was increased concomitantly with glucosuria (Figure 5).

## Discussion

In the current study, we describe two patients who received EMPA treatment for 18 months and 24 months respectively. Our study shows that this treatment was safe and effective in both patients resulting in normalization of ANC and function and yielded clinical improvement characterized by the resolution of oral mucosal and dental infections in both patients and prolonged remission of IBD symptoms in PT1. Moreover, regular GCSF injections were successfully discontinued in PT1 within four weeks of treatment with normal ANC thereafter. Although not evaluated by reliable standardized tools both patients and their families reported improved quality of life following EMPA treatment. Our results are in line with previous case reports (13, 14, 18, 19) and with a recent study reporting the accumulated real-life experience of 112 GSD1b patients treated with EMPA in 24 countries (15). Accordingly, neutrophil counts were improved in the majority of patients which had allowed to stop the regular GCSF injections in 55% of the patients involved in the study. Importantly, similar to PT1, EMPA was effective in achieving prolonged and stable remission of IBD (15).

In this work we monitored 1,5-AG blood levels for both patients. This confirms that the regular EMPA intake lowers 1,5-AG in blood by 5 to 8-fold which results in a new stable baseline already after two weeks of treatment. Of interest the baseline levels before empagliflozin were markedly higher in the younger patient compared with the older girl. This may be explained by the renal tubular dysfunction in the older patient which may impair 1,5-AG reabsorption (Figure 2A).

We elected to give the EMPA to our patients in a single dose, taken once a day. Some reports (10–12) suggest to split the daily dose of EMPA in two daily intakes, but in our patients the single dose appeared to be an effective alternative, which if successful makes it easy to comply to treatment. Given that measuring the concentration of 1,5-AG in blood is not a service that is available in most clinical laboratories, and based on the follow up of our two patients, we recommend measuring 1,5AG both before treatment and 30 days after initiating it. If the level has dropped considerably (4–5-fold) and the clinical improvement is evident, it is possible to continue treatment without further monitoring. When 1,5-AG measurement is not available, empiric treatment with doses ranging between 0.4–0.5 mg/kg/day is acceptable. Since 1,5-AG is the elected biomarker that allows to monitor whether patients are complying to treatment and to best adapt the dosage of EMPA used for each patient, it would be a priority to develop an alternative, more convenient assay for 1,5-AG. This could be for example, the measurement of 1,5-AG (and/or 1,5-AG6P) in dried blood spots, which only needs the withdrawing of a few drops of blood (ideal in very young children) and bypasses the need for sample transportation in dry-ice.

We performed consecutive investigations of urinary excretion of glucose and 1,5-AG in PT2. These studies confirmed that renal clearance of 1,5-AG is related to glucose clearance, as already shown in the treatment of neutropenia in G6PC3-deficient patients with EMPA (16). This may suggest that in GSD1b patients, variations in urinary glucose clearance depending on how well their blood glucose is controlled (and therefore their plasma concentration of glucose) are likely to impact the efficiency with which they clear out 1,5AG from blood and from the body. In this context, it could be interesting to investigate the extent of glucosuria following EMPA in relation with the timing of EMPA and cornstarch takings, for example. It is reported that below the threshold of 160 mg of glucose/dl, the glucose present in the blood is not filtered by the kidney even in the presence of SGLT2-inhibitors (20). In agreement, patients with G6PC3-deficiency (MIM# 611045), that do not have any difficulty in controlling glycemia showed significantly higher glucose excretion rates compared with our GSD1b patient compatible with higher mean blood glucose levels (15, 16). Consequently, G6PC3-deficient patients appear to need significantly lower daily doses of EMPA (0.1–0.2 mg/kg/day) compared with GSD-1b patients to achieve similar 1,5-AG excretion (16).

Since empagliflozin is not approved in children, particular attention should be paid to adverse effects, most specifically hypoglycemia and dehydration. In our study both patients were monitored with continuous capillary glucose devices and had well controlled glucose levels further supporting its safety profile. Moreover, we suggest that early treatment possibly starting in the first years of life may prevent later complications such as IBD and recurrent infections that may trigger metabolic crises and also improve the quality of life of these patients.

## Conclusions

The current study further emphasizes the favorable effect and safety profile of repurposing EMPA, an existing anti-diabetic drug in patients with GSD1b. Additional studies are warranted to better define its long-term efficacy and to identify optimal biomarkers for future surveillance and monitoring.

## Data Availability

The raw data supporting the conclusions of this article will be made available by the authors, without undue reservation.
